# Special Issue on Acoustic Sensors and Their Applications (Vol. 1)

**DOI:** 10.3390/s23187726

**Published:** 2023-09-07

**Authors:** Farook Sattar, Niladri Bihari Puhan, Reza Fazel-Rezai

**Affiliations:** 1 Department of Electrical and Computer Engineering, University of Victoria, Victoria, BC V8P 5C2, Canada; 2 School of Electrical Sciences, Indian Institute of Technology Bhubaneswar, Khordha 752050, India; nbpuhan@iitbbs.ac.in; 3 Mathworks, Natick, MA 01760-2098, USA; rfazelre@mathworks.com; 4 School of Science, Technology, Engineering, and Math, American Public University, Charles Town, WV 64706, USA

Acoustic sensors have been in commercial use for more than 60 years. Acoustic sensing technologies have been studied extensively, and the information, transmission, reception, transformation, processing and application of acoustic signals have been developed, with acoustic sensors as a central focus. An acoustic sensor is a device that converts a sound wave signal into an electrical signal. The design and development of acoustic sensors are very important technological and scientific issues. Acoustic sensors are widely used in industrial, medical and numerous other applications including environmental and health monitoring, chemical and biochemical detection, and signal processing devices. The papers that form this Special Issue cover a variety of approaches and models related to acoustic sensors and their applications.

Liu et al. [1] aimed to fabricate a smart, nickel-based super-alloy bolt with a high-frequency (center frequency: 17.14 MHz) piezoelectric thin-film sensor by radio frequency magnetron sputtering. The proposed high-frequency probe provides a number of advantages over the commercially available piezoelectric probe in terms of a pure and broad frequency spectrum, high temperature tolerance, small preload measurement, repeatability, measured error due to thickness of couplant layer and change in measurement position.

Rong et al. [2] proposed a muffler named infinity tube with an expansion chamber (ITEC) by adopting the transfer matrix method (TMM) for noise control in the ductwork system. A thorough theoretical and numerical study was carried out for this novel sound attenuation device, ITEC, to reduce duct noise. A closed-form expression for the transmission loss of the ITEC device was derived. The advantage of ITEC over IT (infinity tube) in low-frequency noise reduction was demonstrated by comparing the transmission loss between them.

Deng et al. [3] designed an end-to-end speaker recognition system, ResSKNet-SSDP, with an improved feature extraction capability and improved adaptation to the speaker recognition task. The proposed system makes it more suitable for practical application as it is more efficient in terms of the Equal Error Rate (EER) and detection cost function (DCF), and its structure is lightweight with fewer parameters and less interference time compared to many of the existing methods.

Bente et al. [4] proposed broadband air-coupled ultrasound-emitting and -receiving transducers, demonstrating the combined use of a broadband thermoacoustic emitter and an optical microphone as receiver. They showed an initial application for simultaneous determination of thickness and sound velocity of a solid material. The sensor combination presented is simpler to use, and the transducer pair works for all samples that have a thickness resonances (TR) frequency below a critical value of 1 MHz.

Liang et al. [5] presented a secondary phase transform (PHAT) cross-correlation method to improve the performance of the acoustic methods based on cross-correlation for pipeline leakage detection in complex background noise environments, for example, in power plants or other industrial concerns. A sinc interpolation method was then introduced to automatic search for the peak value of the cross-correlation curve. An improved performance of the proposed method is shown for noise suppression and accurate time delay estimation (TDE) compared to the basic cross-correlation method, which can be beneficial in engineering applications.

Yao et al. [6] investigated the capabilities of a commercial AE (Acoustic Emission) sensor in decomposing AE wave modes generated by pencil lead breaks (PLBs) on a thin metal plate incorporating the LAMDA (linear array for modal decomposition and analysis) sensor and finite element analysis (FEA). It is shown that a transverse PLB produces a dominant A0 mode, while a longitudinal PLB produces a combination of A0 and S0 modes. This work can further initiate the application of LAMDA sensors to identify the wave modes generated by the different damage mechanisms in composite panels.

Vetrab and Gosztolya [7] used hybrid HMM/DNN embedding extractor models in computational para-linguistic tasks. The proposed HMM/DNN hybrid acoustic-model-based feature extraction technique was then found efficient at extracting features from different para-linguistic tasks. In order to perform classification through SVM models, different aggregation methods are used to convert acoustic frame-level features into utterance-level features. The proposed scheme can be considered a competitive and resource-efficient approach for various computational para-linguistic tasks.

Liu and Abdulla [8] introduced an improved acoustic power transfer (APT) system by proposing a 3D-printable and cost-efficient piezoelectric transducer equilateral triangular peripheral clamp. This study integrates an impedance matching circuit into the Mason circuit and investigates the impact of fixed constraints on the piezoelectric transducer’s sound pressure and output voltage. The novel findings of this study can aid researchers and practitioners in various fields that employ APT systems to improve their performance in air.

Scheuer and DeCorby [9] applied an ultrasensitive, broadband optomechanical air-coupled ultrasound sensor to investigate the acoustic signals produced by pressurized nitrogen escaping from a variety of small syringes. Harmonically-related MHz-range jet tones were generated by passing pressurized nitrogen gas through a collection of small syringes and shown the extension of the previously established theory to this range. The results from the constructed compact probe for all-optical detection in the MHz range could have practical implications for the non-contact monitoring and detection of early-stage leaks in pressured fluid systems.

In the tenth study, Han et al. [10] developed a sophisticated embedded ultrasonic system to monitor the mechanical properties of ice in real-time and online under various temperature conditions. With the help of the numerical models, the wave propagation in ice was investigated, and the influence of varying temperatures on the wave propagation was also discussed. The proposed system provides a platform to continuously obtain the response of the ice. With this system, the ice properties under specific temperature conditions are then identified based on the intrinsic relationships between the wave propagation velocities and the mechanical properties of ice.

Finally, in the last paper, Jang et al. [11] introduced an electrical equivalent circuit for sonar sensors based on their impedance characteristics by proposing an estimation approach to derive the equivalent circuit. In this study, a particle swarm optimization (PSO) algorithm is employed for parameter estimation of high-degree electrical equivalent circuits. The proposed approach maintained high precision of the derived equivalent circuit even with variations in the number of resonances and the sensor’s impedance characteristics. It is expected to provide accurate estimations of the load characteristics when designing amplifiers for sonar operation.
